# MHC Class I Immunopeptidome: Past, Present, and Future

**DOI:** 10.1016/j.mcpro.2022.100230

**Published:** 2022-04-05

**Authors:** Jonathan W. Yewdell

**Affiliations:** Cellular Biology Section, Laboratory of Viral Diseases, National Institute of Allergy and Infectious Diseases, NIH, Bethesda, Maryland, USA

**Keywords:** immunopeptidome, MHC class I, immunosurveillance, DRiPs, RiboSeq, DRiP, generation of ribosomal product, ER, endoplasmic reticulum, HLA, human leukocyte antigen, IAV, influenza A virus, IFN, interferon, MAP, MHC-associated peptide, MHC, major histocompatibility complex, NP, nucleoprotein, nuORF, novel unannotated ORF, T Ag, tumor antigen, TCR, T-cell receptor

## Abstract

In the 35 years since the revelation that short peptides bound to major histocompatibility complex class I and II molecules are the secret of the major histocompatibility complex–restricted nature of T-cell recognition, there has been enormous progress in characterizing the immunopeptidome, the repertoire of peptide presented for immunosurveillance. Here, the major milestones in the journey are marked, the contribution of proteasome-mediated splicing to the immunopeptidome is discussed, and exciting recent findings relating the immunopeptidome to the translatome revealed by ribosome profiling (RiboSeq) is detailed. Finally, what is needed for continued progress is opined about, which includes the infusion of talented young scientists into the antigen-processing field, currently undergoing a renaissance; thanks in part to the astounding success of T-cell–based cancer immunotherapy.

## Highlights

• Concise history of the discoveries leading to the molecular explanation for the phenomenon of the MHC class I–restricted nature of T-cell recognition.
• Historical review of how MS became a critical technique for defining MHC class I–associated peptides and understanding how peptides are generated from proteins biosynthesized by the antigen-presenting cell.
• Critical review of recent findings linking the translatome to the MHC class I immunopeptidome and the controversy regarding contribution of proteasome-mediated peptide splicing to the immunopeptidome.
• Speculative discussion of the future contributions of MS to understanding the generation of the MHC class I immunopeptidome.

## Graphical Abstract

**Figure:**
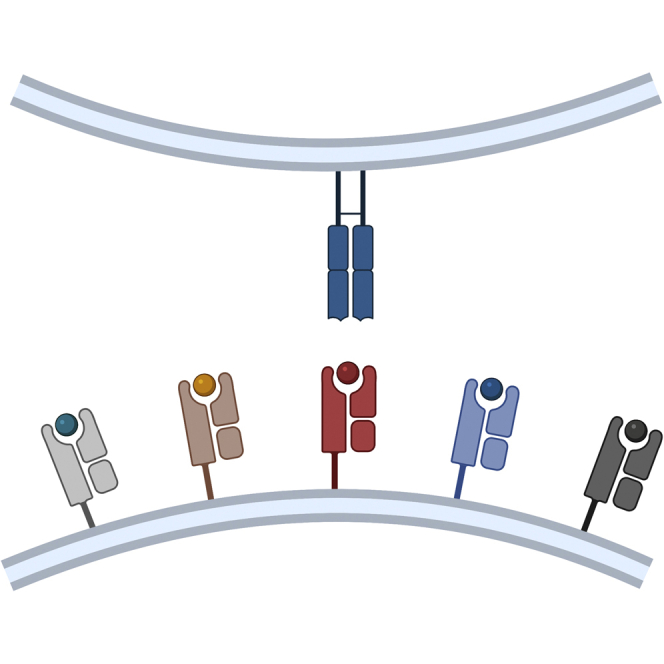

## In Brief

A pioneer in studying CD8+ T-cell immunosurveillance of viruses and tumors reviews the critical contributions of MS-based studies to early, current, and future understanding of how cells generate the MHC class I immunopeptidome: the repertoire of foreign and self-peptides presented by MHC class I molecules for T-cell recognition.

It is a great pleasure to contribute to this remarkable collection of reviews. As a card-carrying immunologist and one of the few non–mass spectrometrists among the August authors, I have tasked myself with succinctly recounting the origins of the immunopeptidome from when it was just a gleam in the eyes of Emil Unanue, Alain Townsend, Stan Nathenson, Hans-Georg Rammensee, Vic Engelhard, and Don Hunt, whose groups each made critical contributions to establishing the basic rules of antigen presentation. But first, a definition: the immunopeptidome (a term I may have coined (1)) is the set of peptides presented by major histocompatibility complex (MHC) molecules on the surface of antigen-presenting cells to enable T-cell immunosurveillance. I will also use this bully pulpit to highlight recent exciting findings linking the translatome to the class I immunopeptidome and finally to speculate about what the future might bring.

## Immunopeptidome Past

The roots of the immunopeptidome lie in studying the genetics of tissue transplantation, which revealed the MHC as a principal locus governing transplant survival, first in mice (H-2) (2), and then in man (human leukocyte antigen [HLA]) (3). Genetic loci led to gene products in the form of MHC class I and II proteins that could be studied first using serum alloantibodies that could be raised in inbred mice and recovered from multiparous women, and subsequently with monoclonal antibodies, which in simpler times, were generously shared gratis widely in the scientific community by their creators (4, 5)

### Setting the Stage: CD4+ T Cells Recognize MHC Class II–Bound Peptides

T-helper cell studies performed using inbred guinea pigs (yes, guinea pigs) provided the first glimpse of the molecular function of MHC molecules (6). Activation of T cells specific for random synthetic peptides known to elicit antibody responses required genetic identity between the MHC of the antigen-presenting cell (macrophages) and the responding T cell, extending findings demonstrating that MHC class II genes controlled the T-helper cell response to synthetic peptides (7). The concept of MHC restriction of T-cell responses fully flowered with Zinkernagel and Doherty's demonstration that T cells would only lyse virus-infected cells that genetically matched the class I genes of the host responding animal (8). Antibodies specific for class II or class I molecules blocked, respectively, helper (9) and killer T-cell (10) recognition of antigen-presenting cells, demonstrating the direct participation of these MHC gene products in the phenomenon of MHC restriction.

The discovery of MHC control of antibody/helper T-cell responses using unstructured random and semirandom synthetic peptides provided a critical clue regarding the molecular explanation for MHC restriction, leading to the proposal that T cells recognize peptides bound to MHC class II molecules (11). The first step in linking artificial synthetic peptides to natural antigens was the demonstration that peptides from “Sigma antigens,” that is, inexpensive purified proteins (that could be obtained from the Sigma Chemical Company) (*e.g.*, hen egg lysozyme, myoglobin, ovalbumin) can induce antibody responses based on their abilities to activate both B cells and T-helper cells (12). Fine-mapping studies established that peptide fragments (13) and synthetic peptides as short as 10 residues could activate T cells induced by immunizing mice with a purified peptide fragment. This paved the way to the initial demonstration of peptide binding to class II molecules (14).

### CD8+ T Cells—Class I in the Spotlight

Because of the basic differences between class I and class II antigen presentation (15), defining relevant class I–associated antigens for CD8+ T-cell recognition required cells expressing source proteins, precluding (though tricks (16) provide a workaround) the use of Sigma antigens. Because of the limited genetic engineering available, it was not possible to identify self-antigens (minor H or tumor antigens [T Ags]), limiting experimental approaches limited to viral proteins. The first viral gene product identified as a class I–restricted antigen was simian virus 40 T Ag, which was shown to be responsible for tumor rejection and T-cell killing of simian virus 40–transformed cells (17, 18, 19, 20). Indeed, the initial evidence (21, 22) regarding the involvement of intracellular degradation to the class I peptidome came from the finding that an unstable fragment of T Ag is fully antigenic. Notably, the same proteins were not immunogenic, which, retrospectively, was probably the first hint for the importance of metabolically stable proteins in T-cell “crosspriming” (23, 24), only firmly established 20 years later (25).

T Ag turned out to be a harbinger for the general phenomenon of CD8+ T-cell recognition of internal viral proteins, which was first inferred from mapping the specificity of influenza A virus (IAV) strain–specific T-cell clones with reassortant IAV, which revealed recognition of a viral polymerase and nucleoprotein (NP) (26, 27). Expression of IAV genes in recombinant vaccinia viruses soon revealed that all IAV gene products can be recognized by CD8+ T cells (28). CD8+ T-cell recognition of cloned IAV NP fragments (29) led to the 1986 discovery that peptides as short as 11 residues sensitized cells for T-cell recognition (30), thus unifying T-cell recognition of class I and class II molecules around the central principle of MHC molecules as peptide receptors that present the products of protein degradation. These seminal findings explained an otherwise puzzling series of earlier observations demonstrating that cyanogen bromide–generated peptide fragments and synthetic peptides as short as 21 residues from viral glycoproteins could stimulate antiviral CD8+ T cells *in vitro* (31, 32, 33, 34). Within a year (35), the crystal structure of HLA-A2 firmly established the molecular basis for the phenomenon of MHC restriction: class I molecules possess a highly polymorphic-binding groove that binds short peptides in a manner that should enable T-cell receptor (TCR) contact with both the peptide and bounding alpha helices. Confirmation of this model came nearly a decade later with the publication of a TCR–MHC class I structure (36), fittingly in the same year that Doherty and Zinkernagel won the Nobel Prize in medicine and physiology for their 1974 landmark article.

### Milestones in Defining Natural MHC I Peptides

The next major step toward defining the immunopeptidome was determining the actual foreign and self-peptides present in the class I–binding groove. A remarkable series of articles (37, 38, 39, 40) used classical biochemical methods to extract low–molecular weight peptides with trifluoroacetic acid and separate them by HPLC with organic solvents. Peptides in fractions were then used to sensitize target cells for CD8+ T-cell lysis or activation. By comparing natural peptide elution with a nested series of peptides containing a known T-cell determinant, or epitope, the natural sequence of the peptide could be inferred. Note that epitope was coined by Niels Jerne to describe antigen surface residues that contact antibodies (41). Since most antigenic peptides are at least partially buried in proteins, endotope or cryptotope (as Jerne originally suggested (41)) is at least as accurate.

This approach revealed that the original IAV NP peptide identified is presented as a 9-mer, which not coincidentally binds class I with the highest affinity among NP-derived peptides. Simultaneously, radiolabeling of vesicular stomatitis virus–infected cells with [^3^H]-amino acids was used in conjunction with HPLC and peptide sequencing to identify a natural viral 8-mer peptide bound to antibody-purified class I molecules (42). Surprisingly, this appears to be the sole published example of using metabolic radiolabeling for peptide identification and characterization; it is well worth revisiting this technique to delineate the kinetics of peptide generation from new *versus* old proteins; though from bitter experience, this is easier written than done!.

These initial studies established the critical point that recovering antigenic peptides requires the expression of class I molecules that bind the peptides with reasonably high affinity (*K*_*D*_ in the neighborhood of 10–100 nM). Further work established that oligopeptides not bound to class I molecules are rapidly degraded (43), though there are exceptions (44). Pragmatically, the most important discovery in this set of articles was the revelation of simple class I allomorph-specific peptide-binding motifs (“anchor residues”) from sequencing of peptide pools recovered from antibody-collected class I molecules (45). This was the first and most critical step to generate the ever-improving algorithms now used to predict class I–binding peptides, an essential step in filtering data to identify valid peptides in contemporary mass spectrometry (MS) characterization of the immunopeptidome.

Just a year after the publication of peptide-binding motifs, the pioneering MS study to characterize host cell class I–bound (46) and class II–bound (47) peptide ligands was published. This was followed by the first MS characterization of viral peptides (48, 49, 50), then the initial description of the virus-infected cell immunopeptidome (51), which revealed major changes to the self-immunopeptidome. This phenomenon largely remains to be mechanistically dissected. It is likely related to findings (52, 53) that viral infections alter translation to favor the generation of ribosomal products (DRiPs) from host mRNAs. In 1996, my colleagues and I proposed that DRiPs, rapidly degraded misfolded nascent proteins, account for the extremely rapid generation of peptides from ostensibly highly stable viral proteins (54). DRiPs appear to be the source of the vast majority of viral peptides, as elegantly shown by MS characterization of the immunopeptidomes of vaccinia and IAV-infected cells (55, 56, 57).

MS played a key role in discovering the presentation of peptides with post-translational modifications (58), which include Ser/Thr phosphorylation (recently shown to be bioinformatically predictable (59)), Asn deamidation during removal of N-linked glycans by peptide–N-glycanase (60), N-terminal acetylation, Cys bound to Cys, glutathione (61), and other S-reactive compounds (61, 62). Cys modification is probably the most prevalent and relevant modification, affecting up to a third of Cys-containing peptides, constituting 5 to 10% of the immunopeptidome (61). Any of these modifications can abrogate recognition by T cells and/or induce T cells specific for the modified peptide. Post-translational peptide modifications include covalent attachment to drugs (63, 64, 65) and other exogenous chemicals (including plant allergens (66)) that can lead to life-threatening autoimmune responses. Indeed, one of the earliest lines of evidence supporting MHC restriction came from studies of cellular modification with trinitrophenol, later shown to be based on peptide modification (67).

Doubtless, the most remarkable post-translational peptide modification discovered is peptide rearrangement *via* protease-catalyzed splicing. Peptide splicing was discovered while searching for a fibroblast growth factor 5 peptide recognized by human antitumor T cells. Puzzlingly, widely separated (40+ residues apart) single Ala substitutions abrogated antigenicity (68). Taking inspiration from the well-characterized splicing of the plant lectin ConA to create the native protein (69), the peptide was revealed to be generated *via* intracellular protein splicing. Within a few months of publication, peptide splicing was reported to occur in the proteasome (70), which provides a ready container for preventing the diffusion of the initial cleavage products. Splicing can even lead to the reversal of the two fragments (71, 72). Since yeast proteasomes splice peptides with similar efficiency as mammalian proteasomes (73), splicing is not evolutionarily adapted for immunosurveillance but likely results from the barrel-like nature of proteasomes. Confirmation from studies on peptide splicing by bacterial and mitochondrial barrel proteases remains to be established, but it is known since 1901 (!) (74) that endoproteases can splice reaction products if present at sufficiently high concentrations (75).

## Immunopeptidome Present: Pressing Issues

### Connecting the Translatome to the Immunopeptidome

By definition, the endogenously presented peptides that constitute the immunopeptidome are synthesized by the antigen-presenting cell’s ribosomes. In 2011, the development of ribosome profiling (76) (RiboSeq) enabled global accounting of the translatome, which defines the potential nonspliced immunopeptidome. The translatome is the ideal database for matching peptides detected by MS since, by minimizing the search space, it optimizes the false discovery rate.

RiboSeq is highly demanding technically and computationally, expensive in time, reagents, and sequencing. But the payback is exquisitely detailed information on exactly what ribosomes are synthesizing, where translation starts, slows, pauses, and stops. Combined with orthogonal techniques (77, 78), it determines the locality of translation of each mRNA (endoplasmic reticulum [ER], cytosol, etc).

To date, only a few studies have related RiboSeq data to peptide generation. Smith *et al.* (79) used a RiboSeq database to identify and validate peptides from endogenous human retroviruses as immunotherapy targets in kidney carcinoma. Erhard *et al.* (80) developed a computational tool (PRICE) that they used to identify noncanonical translation products in a published RiboSeq dataset, some of which are the source of 112 peptides present in an MS immunopeptidome they characterized from the same cell type. This was the first generalization of late 80 s findings by Boon *et al.* that peptides can be generated from sequences lacking the canonical features thought to be necessary for translation (81). Although not included in the immunopeptidome analysis, Erhard *et al.* also found over 500 new noncanonical human cytomegalovirus proteins in a published RiboSeq dataset, all of which are potential peptide sources for antiviral immunosurveillance.

Investigating the immunoribosome hypothesis, which posits a special class of DRiPs generated by a subset of ribosomes (82), Wei *et al*. (83) studied the effects of ribosomal protein knockdown on peptide generation. RiboSeq revealed that RPS28 knockdown increased noncanonical translation of proteins in 3′ and 5' UTRs and proteins initiating with non-AUG codon. Loss of RPS28 also increased HLA-A2 cell surface expression and export from the ER, consistent with an increase in peptide supply from the noncanonical proteins, many of which may represent DRiPs.

Chong *et al.* (84) were the first to experimentally determine the RiboSeq translatome and the MS immunopeptidome on the same sample, a human melanoma cell line. RiboSeq identification of translated proteins expanded the MHC I immunopeptidome by 25%, though all but 56 of the 3606 extra peptides found derived from canonical proteins.

Extending this finding, Ruiz Cuevas *et al.* (85) were the first to correlate the RiboSeq translatome with the MS immunopeptidome and whole-cell proteome. The translatome was generated using a newly devised computational tool (RiboDB) that modestly surpassed PRICE in finding novel translation products. Extensive informatic analysis of three human B-cell lymphoma cell lines revealed that:

1. Fully 85% of translation products (approximately 200,000 for each cell line) are not annotated in the standard human database and constitute the noncanonical translatome. Of these, only 0.08% and 0.36% were detected in the immunopeptidome and proteome, respectively. This is due, in part, to their sevenfold shorter length: the total noncanonical translatome encodes ∼11 million amino acids, compared with 24 million amino acids for the canonical translatome, because of a median length of 42 *versus* 280 residues per protein (this is consistent with RiboSeq-detected noncanonical proteins in other studies (86)). But even for canonical proteins, only 5.51% and 9.11% are detected, respectively, in the immunopeptidome and proteome, the latter emphasizing the current limitations of MS in completely cataloging the proteome.
2. Of noncanonical translatome-defined proteins, half are novel isoforms, proteins that nearly exclusively use a nonannotated start codon, often a non-AUG codon downstream of the annotated start codon. The rest are “cryptic” proteins translated from alternative reading frames of canonical ORFs, “noncoding” ORFs, including introns, 3′ and 5' “UTRs,” intergenic regions, pseudogenes, and other “noncoding” RNAs. More than 70% of cryptic proteins initiate with non-AUG codons, most commonly CUG, but with all near-cognate codons (one base substitution from AUG) well represented.
3. Of 14,498 proteins detected by MS, only 17% are noncanonical proteins, split, 60:28:12, respectively, among “UTRs,” novel isoforms, and frameshifted canonical ORFS. The absence of cryptic proteins from the proteome is based on several factors. Their short size limits the number of peptides that can be detected and likely increases their degradation rate. Their stability is probably also impaired by higher predicted disorder since proteins consisting of less than 80 residues typically need a binding partner for stability. Increased ribosome stalling detected by RiboSeq may also contribute to the decreased stability (87).
4. Of 7045 MHC-associated peptides (MAPs) detected, 7.5% were derived from noncanonical proteins. Note that this is much greater fraction than detected by Chong *et al.*, likely because of the ∼100-fold greater number of RiboSeq reads used to populate the translatome. Of noncanonical MAP source proteins, there is 50:50 split between novel isoforms and cryptic proteins. Canonical and noncanonical peptides had a similar length distribution and predicted class I–binding affinity. As expected, based simply on number of potential peptides in a given protein, longer proteins were a preferred source of both canonical and noncanonical peptides. Importantly, MAP source cryptic proteins were much shorter than canonical source proteins with a median of 49 *versus* 504 residues.
5. For MAP source proteins, levels of canonical transcripts were only 1.4-fold and 2.1-fold higher, respectively, than novel isoform and cryptic protein transcripts. There were also surprisingly slight differences in the translation efficiency of these mRNAs (note that *efficiency* relates only to MAP detection, not MAP quantitation, an important limitation to nearly all global immunopeptidome studies, as discussed later). Critically, per translation event, mRNAs encoding cryptic proteins were fivefold more efficient at generating MAPs. Ribosome stalling, which is greater on such mRNA, may contribute to this increased efficiency, as elegantly shown by Trentini *et al.* (88) using a model substrate.

Ouspenskaia *et al.* (89) determined the RiboSeq translatome for a remarkable 29 human primary and cancer cells. This generated 82,000 annotated and 237,000 novel unannotated ORFs (nuORFs), which were of similar brevity to the cryptic proteins detected by Ruiz Cuevas *et al*., and were also nearly undetected in the whole-cell proteome (0.1% of all tryptic peptides detected). About 6500 peptides from 3300 nuORFs were detected in the immunopeptidome, representing 3.3% of the large (198,000 peptides) 29 cell composite immunopeptidome. Though a tiny fraction of all peptides detected, it is still remarkable that 26 nuORFs encoded the exact peptide detected in the immunopeptidome: the genomic equivalent of minigenes (actually beyond minigenes, which typically possess the minimal peptide plus an initiating Met residue) used to study antigen processing in model systems. Looking specifically at cancer cells, nuORFs contributed to ∼2% of the immunopeptidome. Despite this, in given cancers, for example, melanoma, nuORF peptides augment the tumor-specific “neopeptide” repertoire by 25%, including peptides from tumor-specific gene products as well as tumor-specific mutations in antigenic peptides. This is consistent with nuORFs being a more efficient source of antigenic peptides per translation event, as reported by Ruiz Cuevas *et al.* (85).

Supporting the contribution of noncanonical translation to the immunopeptidome, Erhard *et al.* (90) developed a new informatics pipeline to reanalyze published cancer cell immunopeptidome MS datasets from multiple laboratories consisting of over 400,000 identified peptides. For a selected single human melanoma line, 1563 peptides were derived from cryptic translation products, corresponding to a 4.5% increase in all peptides detected. For all tumors, 12,752 cryptic peptides detected constituted a 2.9% increase in total peptides. Careful analysis of these data led to several remarkable findings.

1. About 18% of peptides from cryptic peptides derive from the COOH terminus of the predicted source protein, with more than half of these within just 10 amino acids from the predicted amino terminus. These peptides should be presented without proteasomal involvement, with amino-terminal residues removed either by cytosolic aminopeptidases or ER-associated aminopeptidases after transport into the ER. Based on this finding, it would be of great interest to reanalyze (91) the peptide dataset tof Milner *et al.* to examine the contribution of cryptic translation to the immunopeptidome in proteasome inhibitor–treated cells.
2. Certain HLA-A alleles demonstrated selective enhancement/reduction in presenting cryptic *versus* canonical peptides, ranging from 43% for HLA-A (136/316; 11:01) to negligible for HLA-B5 (1/7015; 8:01). Across all datasets, 10% of peptides assigned to A03 supertype allomorphs (supertypes are groups of closely related MHC genes) derive from noncanonical translation products compared with less than 2% for A02 supertypes. This could not be facilely attributed to differences in peptide composition and is consistent with potential allomorph-related differences in localized translation of class I molecules to facilitate channeled processing and presentation of cryptic translation products (92).

Bartok *et al.* (93) brilliantly combined RiboSeq and immunopeptidomics to explore how interferon gamma (IFN-γ) signaling–induced Trp deficiency affects the tumor cell immunopeptidome. The resulting reduction in Trp-charged tRNAs decreases protein synthesis because of selective ribosome stalling at Trp codons. Stalling is associated with translational frameshifting, which generates novel peptides, 94 of which were detected in the immunopeptidome of freshly isolated human melanoma cells. A number of these peptides were extensively validated and shown to be expressed in metastases from the same tumor. Several peptides were detected in a tumor from an another MHC-matched patient. One of the peptides was immunogenic for T cells *ex vivo*. This work establishes a new principle for how the tumor-specific peptide repertoire is naturally expanded during the course of an antitumor immune response.

Taken together, the RiboSeq studies demonstrate that while noncanonical translation accounts for much less than one-half of total translation on a molar basis, it accounts for approximately two-thirds of the variety of translation products. Noncanonical proteins are mostly small metabolically unstable proteins, some of which are highly efficient sources of MAP that can be important targets for cancer immunotherapy and possibly autoimmunity as well (94). Surely, some/many of these noncanonical proteins have other biological functions despite their small size and rapid turnover (95, 96). Likely, the noncanonical translatome and derived peptides are greatly altered under infectious stress, which is known to favor noncanonical translation initiation (97). It will be critical to verify and extend findings currently limited to *in vitro* cultured cells to tissues *in vivo*.

### Peptide Splicing: Exception or Rule?

Being the one to suggest splicing to Kenichi Hanada and Jim Yang as a potential mechanism to account for the effects of widely separated amino acid substitution on fibroblast growth factor 5 antigenicity (68), I was still surprised at how rapidly other spliced peptides were reported. It was an even greater shock when Liepe *et al.* (98) reported that spliced peptides account for a third of the immunopeptidome in diversity and a quarter in abundance. Supporting these findings, Faridi *et al.* (99) reported that spliced peptides account for up to 44% of peptides associated with given HLA allomorphs, with a majority of spliced peptides derived from different proteins (trans-splicing).

While proteasome-mediated splicing is a nonstochastic process that greatly favors ligating certain fragments, it is nonetheless an uncommon outcome in detailed studies of proteasome-mediated splicing of synthetic peptides (100, 101), undermining a major contribution of spliced peptides to the immunopeptidome. Further, could simultaneous or sequential proteasome degradation of two different proteins ever occur frequently enough to generate sufficient quantities of trans-spliced peptides for MS detection? This would be possibly plausible for two different DRiPs from highly translated viral proteins (which can each account for 5% or more of the total translation). However, for cellular proteins, it seems nearly impossible for proteins that are not physically associated when delivered to the proteasome for degradation (which, to my knowledge, has yet to be reported).

But data are data, though the devil is always in the details. A critical factor in MS identification of peptides is the false discovery rate, which increases with the number of sequences in the database used to match peptide masses. Without this limitation, MS data could be queried against every possible n-mer peptide (*i.e.*, 20 amino acids at each position [or more accurately 19 amino acids, since Leu and Ile can only be distinguished by special techniques (102)). Indeed, minimizing the relevant peptide database is a major advantage of using RiboSeq in immunopeptidome studies.

Several groups re-examined the reported spliced immunopeptidome and found that 1 to 6% of ostensibly spliced peptides fail to meet more stringent criteria (103, 104). Further detailed informatic analysis, including matches with peptides from cryptic proteins and likelihood of binding to class I molecules, reduced the occurrence of spliced peptides to <1% of the immunopeptidome (90, 105).

The primary difficulty in studying spliced genomic-encoded peptides is the sheer size of the potential peptidome. This is obviated with studying viral peptides, which also provide a much easier method of verifying peptide identity by expressing individual viral proteins, mutating the corresponding gene, or by inducing T cells specific for the peptide. In a comprehensive study of HIV-spliced peptides, Paes *et al.* (106) determined that spliced peptides account for ∼2% of the viral peptidome and that while T cells from HIV-infected individuals can recognize spliced peptides, they do so based on TCR cross-reactivity with one of the unspliced parent peptides. The spliced peptide's lack of apparent intrinsic immunogenicity correlated with the relatively lower abundance of spliced *versus* unspliced peptides.

While further studies are warranted, the present consensus is that spliced peptides constitute perhaps 1 to 3% of the immunopeptidome. Still, if the immunopeptidome is representative of the proteasome-generated peptidome, spliced peptides are likely to have roles beyond immunosurveillance, perhaps even as neurotransmitters, based on the remarkable findings of Margolis *et al.* that a large fraction of DRiPs are degraded by neuronal cell–surface proteasomes, with peptide products having signaling activity (107, 108).

## Immunopeptidome Future

### Sensitivity

Progress in understanding the composition and origin of the immunopeptidome will be heavily dependent on advances in technology. Sooner or (probably) later, greatly increased sensitivity of MS (perhaps on the order of a billion-fold relative to standard experiments today, which routinely start with 10^7^–10^8^ cells) should enable characterizing the immunopeptidome at the level of single cells (109), along with the proteome, degradome, and translatome (which is easier to envisage), with the ultimate goal of studying cells in their natural context in living vertebrates. All this is integral to the broader issue of protein synthesis and degradation at the level of individual cells. Superhigh sensitivity would also enable characterizing the complete immunopeptidomes of antigen-presenting cells engaged in T-cell activation/tolerance in lymph nodes, spleen, and thymus and dissect the contributions of endogenous and exogenous sources of peptides. Increased sensitivity of MS will also enable more precise stable isotope labeling by amino acids in cell culture to accurately measure the contribution of nascent *versus* retired proteins to the immunopeptidomes, where labeling intervals should be on the order of minutes rather than hours to enable detection of peptides from proteins degraded cotranslationally (110) or within minutes of synthesis (111, 112).

### Quantitation

A key to relating translatome and degradome to the immunopeptidome is quantitating the amounts of each peptide detected. At present, this requires using known amounts of synthetic peptides as standards (57, 113). In principle, it should be possible to obtain quantitative data directly during MS analysis. For peptides containing Trp (only ∼10% of the immunopeptidome, because of its being the least abundant amino acid), Trp intrinsic fluorescence (114) should enable direct quantitation after chromatographic separation with a sufficiently sensitive detector. Quantitating other peptides directly will require ingenuity but surely cannot be impossible!

### Cell Biology

To date, the MS-defined immunopeptidome has almost exclusively been used to indirectly infer how peptides are generated. The Admon laboratory has pioneered using the MS immunopeptidome to directly characterize the contribution of cellular processes (*e.g.*, proteasome participation, effects of IFN). Many more of these types of experiments would greatly advance the field. For example, it would be of great interest to examine the repopulation of the immunopeptidome after acid stripping cells to remove class I–bound peptides in the presence and absence of a protein synthesis inhibitor. This should reveal the contribution of DRiPs *versus* retirees at the level of the entire immunopeptidome. Incubation of cells with brefeldin A to characterize peptide class I dissociation would provide novel global insight into real *versus in silico*–predicted class I–binding affinity and how peptide affinity relates to the half-life of MHC I complex. Class I molecules purified from various intracellular compartments could be used to examine how the immunopeptidome matures with transport of class I molecules to the cell surface. Many more such experiments are possible.

### Viral Immunology

To date, only a few studies have exploited the awesome power of MS to characterize the viral immunopeptidome. In a remarkable early MS viral immunopeptidome study, van Els *et al.* (50) reported that HLA-A2 presented a single measles virus peptide in enormous numbers: greater than 10^5^ complexes per virus-infected cell. Such levels are not even achieved by overexpressing preprocessed peptides (115, 116), raising the possibility of virus-evolved peptide overexpression to modulate the CD8+ T-cell or NK cell response.

Extending their prior studies on vaccinia virus, Purcell *et al.* (55) described the quantitative kinetics of IAV peptide generation, discovering seven new peptides in the process, despite the influenza system being intensively investigated previously. The study provides unique information quantitating peptides crosspresented by dendritic cells, explaining the strange immunodominance of the PA_224–232_ peptide, which is presented at extremely low levels on infected cells but is among the most abundant of cross-presented peptides. de Wit *et al.* (117) provided a shining example of the power of MS discovery of viral peptides in reporting 41 mumps virus peptides presented by human class I molecules, six of which were recognized by CD8+ T cells from patients with mumps. If not at present, MS will likely soon become the most time and cost-effective method for identifying viral peptides (118).

Though I have focused this review on MHC class I, I would be remiss to omit class II from the discussion. It has long been known that T_CD4+_ can kill virus-infected cells (119) and that IFN-γ and other cytokines can induce class II expression on nearly all cell types. Class II is constitutively expressed not only by many bone marrow–derived cells but also by a surprising number of other cells, for example, type II pneumocytes, endothelial cells, and gut epithelial cells. In addition to classical endosomal loading of exogenous viral proteins, class II molecules also present endogenous viral peptides (120). Class II–restricted responses to viruses have been greatly understudied generally; specifically, I am unaware of any published MS class II viral immunopeptidome studies. There are clearly opportunities here for basic discoveries in antigen processing/presentation and practical applications in monitoring class II responses, improving vaccines and dealing with viral immune escape.

### Tumor Immunology

This is the one area of the immunopeptidomics that has attracted a reasonable amount of interest from the greater community, thanks to the success of T cell–based immunotherapy and the paucity of tumor-specific targets. Still, so many questions have yet to be addressed. For example, I cannot find a single published study on how radiation treatment alters the MS tumor immunopeptidome, as it certainly must do (121). Ditto for chemotherapeutic agents. Since most tumor antigens must be presented *via* crosspresentation to activate naïve T cells, an obvious issue is the cross-presented immunopeptidome, which is also a critical issue for peripheral tolerance to tissue-specific antigens.

## The Last Word

Many of us generation 1 antigen processingologists have spent much of our careers studying how SIINFEKL and a few other model peptides are generated. This approach has been fruitful, but extending these findings to the greater immunopeptidome is essential. This can only be accomplished *via* MS-based immunopeptidomics. The field should aim at fostering interdisciplinary collaboration between laboratories focused on MS, cell biological aspects of antigen processing, and bioinformatics. Young scientists interested in T-cell immunosurveillance should be encouraged to receive training in at least two of these disciplines. All should attend the 11th and future Antigen Processing and Presentation workshops, where newcomers are not only welcome but cherished.

See you there!

## Conflict of interest

The authors declare no competing interests.
